# Students’ Knowledge Comprehension after Implementation of Live Conventional Demonstration, Video Teaching and Video-Assisted Instruction Methods in Endodontic Practice

**DOI:** 10.22037/iej.2017.39

**Published:** 2017

**Authors:** Nahid Mohammadzadeh Akhlaghi, Zohreh Khalilak, Mehdi Vatanpour, Amirabbas Moshari, Saman Ghaffari, Mohammad Sadegh Namazikhah

**Affiliations:** a *Department of Endodontic, Cranio-Maxillo_Facial Research Center, Dental Branch, Islamic Azad University, Tehran, Iran; *; b *Department of Endodontic, Dental Branch, Islamic Azad University, Tehran, Iran; *; c *Private Practice, Tehran, Iran; *; d * Former Chair of Endodontic Department, University of Southern California, Los Angeles, California, USA*

**Keywords:** Conventional Education, Endodontic Treatment, Knowledge, Performance, Video-Assisted Clinical Instruction

## Abstract

**Introduction:**

Video-assisted clinical instruction (VACID) has been found to be a beneficial teaching tool for various fields in dentistry. The aim of this interventional study was to compare the efficacy of live conventional demonstration (CD), video teaching, and VACID (video with explanation) methods in teaching of root canal treatment to undergraduate dental students.

**Methods and Materials:**

Forty-two undergraduate senior dental students participated in this study. The students experienced this course for the first time and were randomly divided into three groups (*n*=14). Group A attended live CD on a patient; group B watched a professionally produced demonstration video without any verbal explanation during 1 h; and finally group C watched the same video alongside live explanation by a mentor during the 1.5 h (VACID). The whole process was performed by an experienced endodontist on maxillary central incisors. All of The students carried out a multiple choice question exam to evaluate their comprehension. The mean score of the experimental groups were compared using ANOVA test and multiple comparisons were carried out with Tamhane test. The level of significance was set at 0.05.

**Results:**

There was significant difference among three groups according to the ANOVA test (*P*<0.05). Group VACID had the highest mean scores. There was significant difference between the groups VACID and VT (*P*=0.011); no significant differences were found in other inter-group comparisons.

**Conclusion:**

According to the results, VACID may improve the quality of endodontic training in undergraduate dental students.

## Introduction

Live conventional demonstration (CD) has always been the traditional method of teaching endodontics to dental students where the mentor performs the step-by-step treatment in the presence of students [1]. This method has shown to be effective in increasing the self-confidence of students, their communication skills and better perception of clinical procedures [2]. Improving this process is beneficial in reducing the risk of procedural errors. Several techniques have been suggested for improving the teaching process such as recording the treatment steps for future reference and on-screen demonstration review of the treatment process using an intraoral camera (video teaching-VT). Multimedia instruction is a well-established means of instructional delivery and is often used to complement or blend with traditional didactic elements or replace other traditional teaching methods altogether [3, 4].

Video-assisted clinical instruction in dentistry (VACID) is an educational tool that uses video-images to complement dental education [1]. The use of mechanical devices as educational tools emerged in the 1950s with Skinner’s “teaching machine”, a machine that allows students to respond to the questions [5]. The superiority of VACID over traditional teaching for education of clinical skills has been reported in several studies. Educational movies have been used to instruct the patients and students in a short time [6]. It has the advantages of storing large amount of information, providing continuity in the learning process, and reducing stress during teaching [7]. Educational movies also help learning of both the technical and clinical skills [8]. Moreover, benefits of this technique in improving technical skills and simulation of clinical setting have been confirmed. Also, students have shown high acceptability towards this technique which is probably attributed to the better observation of clinical procedures and improved interpretation of details [1]. VACID has been found beneficial for teaching in dentistry for a wide range of areas, including training in mechanical or procedural skills [9, 10].

Regarding to the advantages of electronic learning and its capabilities in medical teaching, it is progressively becoming more common in dental education. However, it could not replace emotional and human inter-relationships between the teacher and students. In addition e-learning eliminates face to face communication through the course of training [11]. One of the most important disadvantages of e-learning and distance education is elimination of virtual active communication between trainers and trainees; therefore, many studies suggest the use of face-to-face method instead of electronic and multimedia techniques [12].

The purpose of this study was to compare the efficacy of live conventional demonstration (CD), video teaching (VT), and video-assisted clinical instruction in dentistry (VACID) in teaching of the root canal treatment procedures for undergraduate dental students.

## Materials and Methods

Forty-two undergraduate senior dental students consensually participated in this interventional study in Endodontic Department of Islamic Azad University, Dental Branch, Tehran, Iran. The students experienced this course for the first time. They were randomly divided into three groups by simple randomization technique, consisting of 14 students in each group: group A; attended in a live CD on a patient, group B watched a professionally produced live demonstration video without any explanation during 1 h (VT) and group C watched, the same video alongside some explanation to the students during 1.5 h (VACID).

Each group members were taught charting, pre-procedural preparation techniques and other essential trainings for endodontic treatment, including access cavity preparation, root canal preparation and obturation of the canal.

All of the demonstrations (CD, VT and VACID) were performed by an endodontist on maxillary central incisors of the patients, in the same day. 

Assessment of students’ learning was carried out with a multiple-choice question exam. This exam consisted of all parts of educated items and was designed by the endodontic expert panel. All of the students attended in an exam with 50 multiple-choice questions on the principles of charting, patient preparation and root canal treatment of anterior and premolar teeth. Each question had one objectively correct answer. 

The mean of the score of experimental groups was compared with each other using ANOVA test and multiple comparisons were carried out with Tamhane test. The level of significance was set at 0.05. 

## Results

There were significant differences among three groups according to the ANOVA test (*P*<0.05). VACID group had the highest mean of scores. Although there was significant difference between the groups VACID and VT (*P*=0.011), the differences between the groups VT and CD and between the groups CD and VACID were not significant (Table 1).

**Table 1:** Mean (SD) of the exam scores in each group. Significant difference was present among 3 groups

	**Mean (SD)**	**Min**	**Max**
**Conventional demonstration**	36.90 (5.56)	29.17	47.91
**Video teaching**	31.82 (6.36)	12.50	45.83
**Video-assisted clinical instruction**	40.92 (4.05)	33.33	45.83

## Discussion

The aim of this study was to compare three methods of teaching (CD, VT and VACID) root canal treatment procedures to undergraduate dental students in Endodontic Department of Islamic Azad University, Dental branch, Tehran, Iran.

Results of this study showed that the VACID group had the highest mean score and the VT group had the least ones. The difference between the groups VACID and VT was significant (*P*=0.011). However, the differences between the groups CD and VT or between the groups CD and VACID were not significant.

Endodontic training for the undergraduate students covers the whole process of patient management, charting, diagnosis, treatment planning, and access cavity preparation, working length determination, root canal preparation and root canal obturation. Live demonstrations have long been the main mean of educating the students in most of dental fields including endodontics. Number of student’s involved, patient selection for teaching the basic skills, other patient related difficulties and the limited field of view in endodontic treatments may bring interferences in the course of learning for the novice students. Difficulties of these kinds convinced the educators to provide instructional videos (including step- by-step demonstration of root canal treatment procedures) for students. Also instruction videos have the advantage of probable reviewing by the student any time after the first teaching.

Education methods like videos are useful methods to improve the level of learning in dental students especially in the field of endodontics, but it seems that CD in conjunction with VT (*aka*. VACID) may result in more improves of students’ learning. This is in agreement with Kalwitzki *et al.* [8] who insisted on VACID according to the students satisfaction. Virdi and Sood [13] used VT in conjunction with CD and live lecture to develop a five step education method in the field of pediatric dentistry and concluded that these elements could speed up the process of learning and made more satisfactory for the students.

Different fields of dental education have used various methods of education. Aragon and Zibrosky [14] showed that adding videos to fixed prosthodontic training may help skill learning. Also Kalwatzky *et al. *[15] compared lecture and VT in developing the communication pattern in pediatric dentistry.

A systematic review on the effect of computer-assisted learning in endodontic education found that both traditional and newer methods have the same effectiveness, but more studies are required [16].

Although Moazami *et al. *[17], found virtual method more effective than the traditional method in teaching dental skills in dental students, other recent studies stated that both methods are similarly effective in teaching endodontic, dental prosthesis and other skills in dental educations [18, 19]. 

Educational videos may eliminate the intervention of patient management issues and the limited field of view in the process of learning the endodontic procedure. In this study, explanation during watching the video movie (VACID) had the benefits of both teaching methods. Therefore it might increase the benefits of students’ perceptions and significantly improve the exam scores after the training process.

This method of teaching may be accomplished with watching the video for more than one time and may improve the learning of endodontics in dental students. Also other methods of assessments like clinical/preclinical assessments may help in determining the best possible training approach in endodontics.

In this study the higher mean scores of group VACID could be attributed to the positive effects of adding a comprehensive live explanation to the instruction video. It omits the stresses induced by patient related factors for both the students and the teacher and therefore increasing the benefits of the presence a real instructor to the students. So this method could be considered as having very positive effects on teaching endodontic skills for dental students.

To our knowledge, this study is the first study in the field of endodontic training, which compared different clinical teaching methods for undergraduate dental students. A limitation of this study may be that only students’ comprehension was assessed and not their active application. However, comprehension and ability to recall is the first step towards complete learning of a new skill.

## Conclusion

According to the results of this study, it’s suggested that using video movies in conjunction with the explanation may improve the quality of endodontic training in undergraduate dental students. Further studies are advised in this field.
